# Effect of Shear Stress on Isothermal Crystallization Behavior of CaO-Al_2_O_3_-SiO_2_-Na_2_O-CaF_2_ Slags

**DOI:** 10.3390/ma11071085

**Published:** 2018-06-26

**Authors:** Shaopeng Gu, Guanghua Wen, Zequan Ding, Ping Tang, Qiang Liu

**Affiliations:** 1 College of Materials Science and Engineering, Chongqing University, Chongqing 400044, China; spgu@cqu.edu.cn (S.G.); zqding126@126.com (Z.D.); tping@cqu.edu.cn (P.T.); liu.qiang@cqu.edu.cn (Q.L.); 2 The State Key Laboratory of Mechanical Transmission, College of Materials Science and Engineering, Chongqing University, Chongqing 400044, China

**Keywords:** mold fluxes, crystallization property, shear stress, quantitative analysis, constant temperature

## Abstract

How to coordinate the contradiction between lubrication and heat transfer in the peritectic steel casting process is the key technical difficulty in preparing mold fluxes. The mold fluxes that are required for casting are subjected to the shear stress generated by mold oscillation and slab movement, which affects the crystallization performance of slags. The quantitative effect of slags’ crystallization performance by shear stress is studied to develop a low-basicity and high-crystallization mold flux to solve the above problem. The results show that the crystallization kinetic condition is promoted, and the crystallization activation energy is reduced by the shear stress, which leads to an increase in the crystallization temperature. Concurrently, the crystal size is reduced. However, the shear stress has no effect on the crystalline phase. The influence of different shear stresses on the crystallization ability of molten slags is related to the crystal nucleation and growth mechanisms. The crystalline fraction of the slag films at 300 rpm (69 s^−1^) is 44.7%, which is an increase of 17.7% compared with the crystalline fraction of the slag films at 200 rpm (46 s^−1^). Moreover, the shear stress has little effect on the lubricating properties of the mold fluxes, although the crystallization ability is promoted by the agitation.

## 1. Introduction

Mold fluxes containing various fluxes and carbon are functional materials based on silicate. They play an important role in ensuring the smoothness of the continuous casting process and the quality of steel in the continuous casting process [1,2]. Mold fluxes, which sit on top of the mold, cover the molten steel and provide thermal insulation to prevent the steel from freezing, prevent oxidation of the steel surface, and absorb non-metallic inclusions from the molten steel. Mold fluxes in the lower half of the mold infiltrate the mold/shell channel, regulate the heat transfer between the shell and the mold, and lubricate the newly formed shell, which are two of the most important effects among all of their functions.

Surface defects such as longitudinal cracks in the continuous casting of peritectic steel slabs, with the carbon content being 0.09–0.16%, can be formed due to the violent release of stress caused by the peritectic reaction and peritectic phase transformation. Controlling the heat transfer from the shell to the mold can solve this problem [3]. Presently, the main method involves regulating the crystallization behavior of mold fluxes, such as increasing the basicity of mold fluxes to produce many crystals that control the horizontal heat transfer over the meniscus region. However, lubrication of the shell can be deteriorated by the high-alkalinity mold fluxes, and it can even enhance the possibility of breakout by sticking [4,5,6]. Therefore, the low-basicity and high-crystallization mold fluxes become the ideal slags for the casting of peritectic steels.

**Figure 1:**
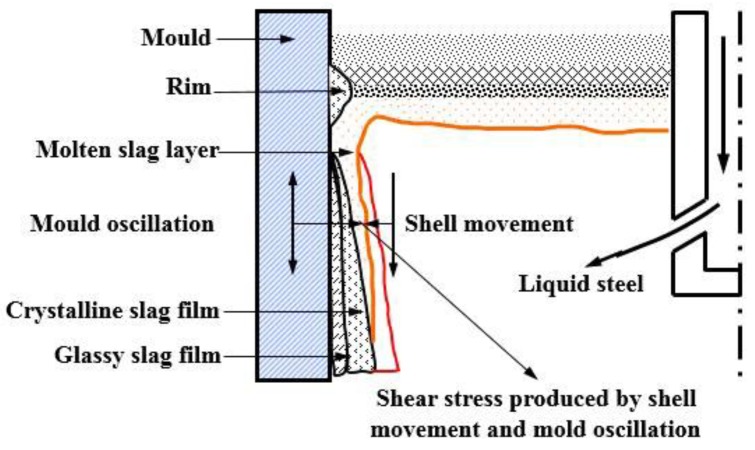
Schematic diagram of mold fluxes between mold and shell.

During the continuous casting process of steel, mold fluxes infiltrating into the shell-mold gap are subjected to shear stress from the mold oscillation and the slab movement [7,8,9,10,11,12,13], as shown in Figure 1. The crystallization of mold fluxes is affected by the shear stress. Therefore, the authors of the current study hope to develop low-basicity and high-crystallizing mold fluxes through shear stresses to overcome the contradiction between the lubrication and heat transfer in the peritectic steel casting process, so that the quality of casting slabs can be improved, and sequence casting can be carried out. Saito et al. [14] studied the effect of shear stress fields exerted by agitating liquid slag using a Pt-Rh rod on the crystallization of CaO-SiO_2_-R_2_O (R = Li, Na, or K) melts. The crystallization temperature was determined using a technique that was based on the difference in the electric permittivity of the ionic liquid and solid. The results showed that the crystallization temperatures of the molten slags were increased by agitation; Harada et al. [15,16] investigated the effect of agitation on the crystallization of CaO-SiO_2_-CaF_2_ and CaO-SiO_2_-CaF_2_-RO (R = Mg, or Sr) melts with the same method. The results showed that the influence of agitation on the crystallization was related to crystal growth and nucleation mechanisms. The crystallization of dendritic CaO·SiO_2_ crystals was strongly affected by agitation, while the crystallization of faceted Ca_4_Si_2_O_7_F_2_ crystals showed little dependence on agitation. Meanwhile, Li et al. [17] studied the effect of shear stress fields on the crystallization performance of CaO-Al_2_O_3_-SiO_2_-Na_2_O-CaF_2_ multi-component slag systems. The results pointed out that the effect of shear stress fields on the crystallization of mold fluxes was achieved by affecting the crystallization kinetics, and the quantitative effect of shear stress exerted by a rotating molybdenum rod at a speed of 100 rpm on the crystallization ability of the mold flux was obtained by image analysis.

The above studies indicate that the shear stress fields can promote the crystallization of slags. However, the experiments of these researchers were carried out during cooling. The temperature change has a great influence on the crystallization of slags. Second, the selected shear rates of those experiments were low (0–21.6 s^−1^) (during the experiments, the shear stress was generated by the rotating rotor, and it was used to simulate the shear stress experienced in the actual continuous casting process. The relationship between the shear rate and the rotational speed of the rotor is shown in Equation (1) [16,18]).

(1) $$\gamma =\left(\frac{\pi N}{15}\right)\left(\frac{1}{1-{\left(r_{a}/r_{b}\right)}^{2}}\right)$$

where *γ*, *N*, *r_a_*, and *r_b_* are the shear rate, rotating speed, radius of the rotor, and inner radius of the crucible, respectively.

The maximum speed selected in the above studies was 100 rpm (21.6 s^−1^). Watanabe et al. [18] pointed out that during the continuous casting process of steel, the shear rate to which mold fluxes were subjected between the mold and the strand ranged from 20 s^−1^ to 125 s^−1^, corresponding to rotating speeds between 87–513 rpm. According to Equation (1), it differed greatly with the selected value in experiments by the above-mentioned researchers. Therefore, it is greatly significant that the influence of shear stress according to the actual casting process on crystallization should be studied. Finally, the previous studies were limited to qualitative study on the crystallization behavior by the shear stress. Although Li et al. [17] found a quantitative relationship between the shear stress and the crystallization performance, the selected rotational speed was constant, and the actual shear stress during the continuous casting process was a changing value. More importantly, the scanning electron microscope (SEM), which was the image analysis method used by Li et al. to analyze the crystalline fraction on a small point of the slag film, does not reflect the crystalline fraction of the entire slag film [17]. Thus, the quantitative relationship between the different shear rates and the content of crystal produced by the agitation in the slag film cannot be established. Therefore, it is worthwhile to develop a better method to quantify the crystalline fraction of the mold fluxes under a constant temperature.

The quantitative influence of the shear stress on the crystallization of mold powders was carried out under a constant temperature of 9 °C above the liquidus temperature and with rotational speeds of 100 rpm, 200 rpm, 300 rpm, and 400 rpm, respectively. The aim of this study is to provide a theoretical basis for preparing the low-basicity and high-crystallization mold fluxes that were applied in the casting process of peritectic steel.

## 2. Experimental Section

### 2.1. Material Preparation

Reagent grade powders of CaO (>99.5 mass percent), SiO_2_ (>99.5 mass percent), Al_2_O_3_ (>99.5 mass percent), Na_2_CO_3_ (>99.5 mass percent), and CaF_2_ (>99 mass percent) were used as raw materials. The compositions of the mold flux used in the present study are given in Table 1. According to the proportions shown in Table 1, several 250 g samples were prepared, mixed, and calcined in a muffle furnace at 373 K (100 °C) to remove moisture. One calcined 250 g sample was placed in a silicon-molybdenum electric furnace and kept at 1400 °C for 30 min to homogenize its chemical composition. Then, the molten slags were quenched by pouring in cold water, dried, crushed, grounded, and then sieved using a 200-mesh screen to afford sample powders for the hemispherical spot method and hot thermal couple technique. Another calcined 250 g sample was placed in a silicon-molybdenum furnace (Xinya Automation Equipment Co., Ltd. , Chongqing, China) heated to 1300 °C, and held for 20 min. Then, the molten slags were stirred for 5 min, 10 min, 15 min, 20 min, 25 min, and 30 min using a stirrer at 100 rpm, respectively. A preheated steel bar (Φ 15 mm × 80 mm) was stuck into the molten slag for 10 s and quickly extracted for quenching, and then drying. The remaining slags were cooled naturally. The above procedure was repeated while changing the rotating speed to 200 rpm, 300 rpm, and 400 rpm to obtain samples with different stirring intensities and times, respectively. Finally, the sample with no agitation (0 rpm) was also obtained using the same method. Some water-quenched and naturally cooled samples with different stirring intensities and times were crushed, grounded, and sieved using a 200-mesh screen for differential scanning calorimetry (DSC) (NETZSCH-Gerätebau GmbH, Selb, Germany) and X-ray diffraction (XRD) (Shimadzu, Kyoto, Japan) experiments, while others were polished for SEM (TESCAN, Brno, Česká) experiments.

### 2.2. Experimental Methods

**Figure 2:**
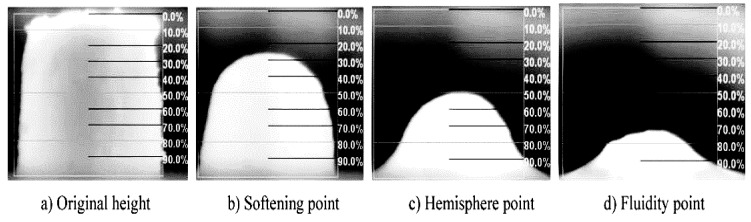
Schematic diagram of the hemispherical point method. (**a**) Original height; (**b**) softening point (3/4 of original height); (**c**) hemisphere point (2/4 of original height); (**d**) fluidity point (1/4 of original height).

The hemispherical point method was used to test the flow temperature (estimating the liquidus temperature) of mold fluxes [19]. The schematic diagram is shown in Figure 2. Flow temperature is defined as the temperature at which the height of slag column became ¼ of the height of the original slag column in the silicon-molybdenum electric furnace in the heating process. Some researchers [3,20] believed that the flow temperature is approximately equivalent to the liquidus temperature at which mold fluxes are completely melted.

The slags were analyzed with the hot thermal couple technique at a heating rate of 15 °C/s to 1300 °C, which was 9 °C higher than the liquidus temperature (to ensure that the slags are in a completely homogeneous state). The temperature was maintained for 5 min to observe whether crystals could form in the molten slags. Some researchers [21] showed that the residence time of mold powders in the mold was approximately 5 min.

**Figure 3:**
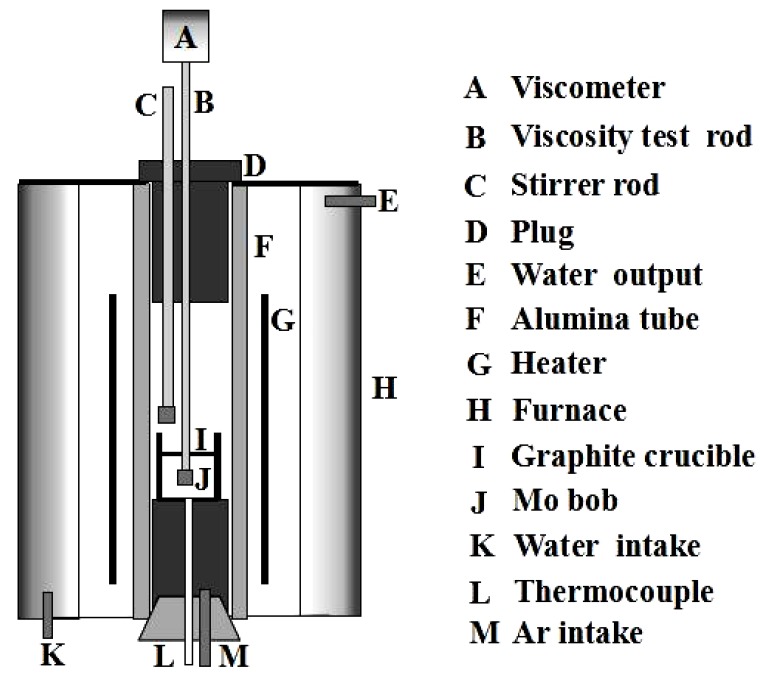
Schematic diagram of experimental apparatus.

The viscosity–temperature curve of the mold fluxes was measured by a rotating cylinder method through the steady-state and dynamic measurements of the viscosity (BROOKFIELD, Middleboro, MA, USA) [22], and the break temperature was obtained by a tangential method through the viscosity–temperature curve (the break temperature is the point where the viscosity rises abruptly, meaning that the liquid-phase lubrication disappears, and the solid-phase lubrication begins at a certain cooling rate). The schematic diagram of the experimental apparatus is shown in Figure 3. The steady method was to put 250 g samples into the graphite crucible (Φ 75 mm × 100 mm), which was placed in a silicon molybdenum furnace at 1350 °C for 30 min to homogenize its chemical composition; then, the viscosity of molten slag was continuously measured during the cooling process at the rate of 5 K/min to obtain the viscosity–temperature curve. Regarding the balance method, the viscosity of the molten slag was measured at different temperatures while holding for 10 min during the cooling process to obtain the viscosity–temperature curve. The viscosity–temperature curve of molten slag was measured by the balance method in the current study. During the cooling process, the viscosity was measured at a certain temperature after the molten slags were stirred for 5 min at the speed of 100 rpm with the stirring rod, as shown in Figure 3. The viscosity at different temperatures was measured until the viscosity–temperature curve was obtained. The above procedure was repeated by changing the rotational speed to 200 rpm, 300 rpm, and 400 rpm respectively, so the viscosity–temperature curves under different shear stresses were obtained. Then, the viscosity–temperature curve with no agitation (0 rpm) by the balance method was obtained, and it was compared with the viscosity–temperature curves of molten slag with different shear stresses.

The thermal analysis method was used to analyze the enthalpy of samples that were under different stirring intensities and times in the argon atmosphere, followed by the crystalline fraction in the slag being calculated based on the enthalpy [23,24]. Approximately 10 mg of mold powder was heated from room temperature to 1000 °C with a heating rate of 20 °C min^−1^. Meanwhile, the data for releasing heat was recorded during the heating process.

XRD and SEM were used to analyze the phase compositions and the microscopic morphology of the samples under the different stirring strengths, respectively.

## 3. Results and Discussion

### 3.1. State of Slags at 1300 °C

**Figure 4:**
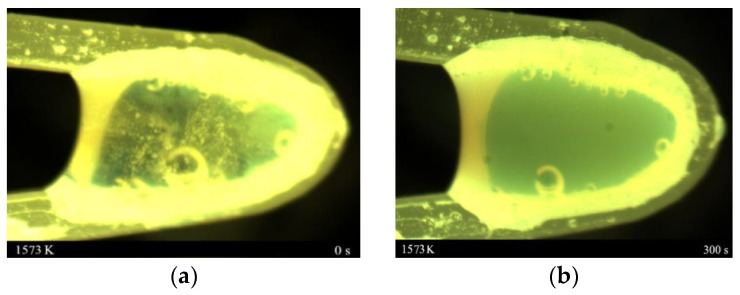
The state of sample at 1300 °C (**a**) held for 0 min; (**b**) held for 5 min.

The liquidus temperature of the mold fluxes measured by the hemispherical point method was 1291 °C. Figure 4a,b show the molten slag state diagrams of the sample were first heated to 1300 °C, and were then held for 5 min by the hot thermal couple technique, respectively. It can be seen from Figure 4a,b that some bubbles are in the molten slags, which are in a completely molten state, and no crystals formed after 5 min at 1300 °C. It remains in a molten state.

### 3.2. Quantitative Effect of Shear Stress on the Crystallization Property of Mold Fluxes

The crystalline fraction of the slags stirred at 1300 °C for 5 min under different rotating speeds is listed in Table 2. It is found from Table 2 that the crystalline fraction in the slag was 2.7% with no agitation. The reason is that the temperature of the steel rod that was preheated for 2 min was lower than the temperature of the molten slag, and the molten slag was cooled naturally in the process when the slag was extracted from the silicon molybdenum furnace with the steel rod. Thus, a small amount of crystals was produced in the slight supercooled slag film [25]. When the rotational speed was 400 rpm, the crystalline fraction in the slag film was 47.6%. The crystalline fraction increased with the increasing rotational speed. Comparing the increment of crystalline fraction from 100 rpm to 300 rpm, the increment increased as the rotating speed increased. Conversely, the increment decreased as the rotating speed decreased from 300 rpm to 400 rpm. The increment of crystalline fraction reached the maximum value when the rotational speed changed from 200 rpm to 300 rpm. The reason might be that the limiting factor of the slag crystallization ability was the ions’ diffusion process at the initial stage. The agitation increased the kinetic energy of the ions, and made the crystal easier to grow. Accompanying the continual increase of the rotational speed, the limiting factor of the slag crystallization ability changed from the diffusion-controlled to the interface reaction-controlled factor. Therefore, the increment of the crystalline fraction slowed due to the agitation having little impact on the interface reaction on the surface of the nucleus.

### 3.3. Effect of Shear Stress on Crystalline Phase of Mold Fluxes

**Figure 5:**
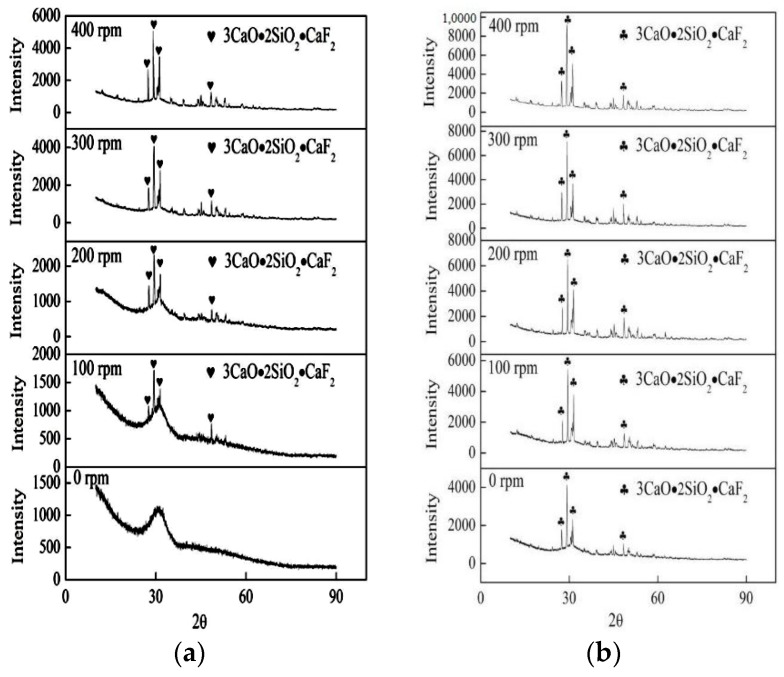
Crystalline phases of the slag films under different rotating speeds for 5 min (**a**) obtained in water quenched at 1300 °C; (**b**) obtained during natural cooling to room temperature.

The crystalline phases of samples water quenched in high temperature and cooled to room temperature with different stirring intensities for 5 min are shown in Figure 5a,b respectively. Figure 5a shows that the slag film was in a glassy phase without stirring. Then, some crystalline phases began to precipitate in the slag film under stirring conditions, and the crystalline fraction increased when the stirring intensified. The cuspidine phase was the only crystal phase in the slag film. Meanwhile, it could be found in Figure 5b that only the same crystalline phases existed in the slag film with different rotating speeds, which is consistent with the crystal phase of the water-quenched samples. This indicates that the crystalline phase was not affected by the shear stress fields. However, the crystalline fraction increased. The degree of supercooling of molten slag gradually increased, and the crystal growth time continued to increase during the process of slag films being naturally cooled to room temperature. Therefore, more crystals could be produced. Meanwhile, the internal arrangement of grains was relatively regular, and the grain size became larger after 8 h of crystal growth, which increased the crystal’s integrity.

### 3.4. Effect of Shear Stress on Micromorphology of Mold Fluxes

**Figure 6:**
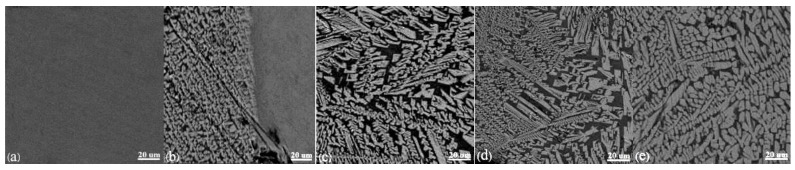
The micromorphology of water-quenched slag films under different stirring intensities at 1573 K for 5 min (**a**) 0 rpm; (**b**) 100 rpm; (**c**) 200 rpm; (**d**) 300 rpm; and (**e**) 400 rpm.

**Figure 7:**
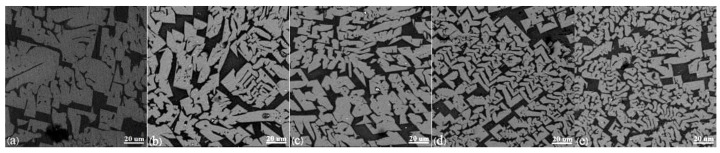
The micromorphology of natural cooled samples stirred for 5 min at 1564 K with different agitation speeds (**a**) 0 rpm; (**b**) 100 rpm; (**c**) 200 rpm; (**d**) 300 rpm; and (**e**) 400 rpm.

Figure 6 indicates the micromorphology of slag films quenched by water under different stirring intensities at 1300 °C for 5 min, and the micromorphology of natural cooled samples stirred for 5 min with different stirring speeds can be found in Figure 7. The rotational speeds shown in Figure 6a through Figure 6e and Figure 7a through Figure 7e are 0 rpm, 100 rpm, 200 rpm, 300 rpm, and 400 rpm, respectively. Among them, the slag film surface in Figure 6a is smooth and bright; the pure glassy phase is proved by the analysis of XRD data. Figure 6b through Figure 6e demonstrate that the slag films surface are crystals, and as the rotational speed increases, the crystalline fraction increases. It is the same as the trend of data in Table 2. However, the grain size decreases gradually. The reason is that the rotor breaks up a portion of the grown dendrites to reduce the grain size. Concurrently, the collision probability is increased, as is the kinetic energy between the crystals and the crystals from collision, and the rotor and the crucible wall from stirring. Therefore, the crystals more easily crash, and the fragment parts will become a secondary nucleation site, producing more tiny crystals. Additionally, the relative flow rate between the crystals and the molten slag is increased by the higher rotating speed, so the ions involved in the crystallization could have difficulty adhering to the grain surface, which would inhibit the crystal growth [26]. Figure 6e shows that some faceted crystals are precipitated. Saito et al. [14] pointed out that the morphological variations of crystals might be caused by agitation, and the growth of faceted morphology crystals was controlled by reaction at the crystal–melt interface. Comparing Figure 7a with Figure 6a, faceted and columnar grains are precipitated in the surface of the slag film, and they are also the cuspidine phase combined with the XRD data. It further verifies that the shear stress fields have no effect on the crystalline phases. Comparing Figure 7b through Figure 7e with Figure 7a, it can be found that the grain size decreases gradually; the maximum decrement of grain size can reach 61% using the software of Image-Pro Plus, and the content of the faceted grains does not change significantly. The reason might be that the limiting factor of the faceted grains is the interfacial reaction, which is little affected by the agitation. Harada et al. [15,16] pointed out that the effect of shear stress on the crystallization of mold fluxes was related to the crystal growth and nucleation mechanisms.

### 3.5. Effect of Shear Stress on Crystallization Kinetics of Mold Fluxes

#### 3.5.1. Effect of Shear Stress on the Crystallization Mechanisms of Mold Fluxes

The kinetics of mold flux isothermal crystallization involving nucleation and growth are analyzed through the Johnson–Mehl–Avrami (JMA) model [27,28,29,30]. According to the JMA model, the volume fraction of crystals *X*(*t*) is given by:

(2) $$X(t)=1-\exp(-kt^{n})$$

where *X*(*t*) is the relative degree of crystallinity at a given time *t*, including the incubation time, *n* is the Avrami exponent, which is associated with the nucleation and growth mechanism, and *k* is the effective crystallization rate constant, which is dependent on the temperature and the rate of nucleation and crystal growth.

The process of crystals precipitated from mold fluxes is accompanied by significant heat release, which can be measured by the DSC technique. Since the rate of the heat release is proportional to the rate of the crystallization, the relative degree of crystallinity *X(t)* can be obtained according to the following equation:

(3) $$X(t)=\frac{\Delta H_{t}}{\Delta H_{total}}=\frac{\int_{0}^{t}(dH_{c}/d_{t})dt}{\int_{0}^{\infty }(dH_{c}/d_{t})dt}$$

where Δ*H_t_* is the enthalpy as a function of the ensuing time from the initial to a given crystallization time, and Δ*H_total_* is the total enthalpy reached at the end of the isothermal crystallization process.

The values of *n* and *k* can be obtained by fitting the double logarithmic form as follows:

(4) ln{−ln[1−x(t)]}=lnk+nlnt

**Figure 8:**
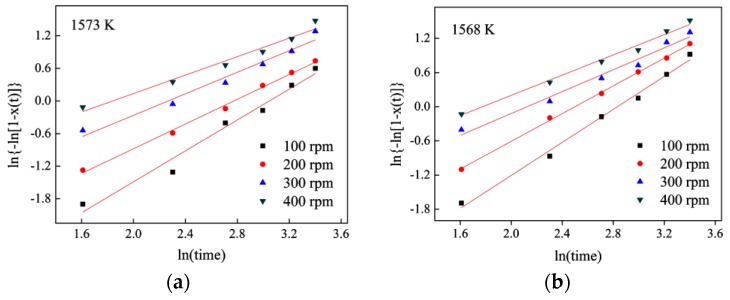
Plots of ln{−ln[1 − *X*(*t*)]} versus ln*t* for isothermal crystallization of mold fluxes under different rotating speeds for 5 min (**a**)1573 K; (**b**) 1568 K.

The double logarithmic plots of ln{−ln[1 − *X*(*t*)]} versus ln*t* for slags at 1573 K and 1568 K are shown in Figure 8a,b respectively. Values of the Avrami exponent *n* and effective crystallization rate constant *k* can be obtained from the slope and the intersection of the plots, as summarized in Table 3.

Table 4 shows the values of n for various crystallization modes. It is well-known that the Avrami exponent *n* is an effective kinetic parameter to determine both the nucleation mode and the dimensionality of crystal growth for crystallization.

(5) $$n=n_{d}+n_{n}$$

where *n_d_* represents the dimensionality of the crystal growth (such as one, two, and three dimensionality), and *n_n_* is the time dependence of the nucleation. It should be noted that *n_n_* can be either 0 or 1, corresponding to the instantaneous nucleation or homogeneous nucleation at a constant rate, respectively, as can be seen in Table 4.

Table 3 shows that the average values of the Avrami exponent at a rotating speed of 100 rpm is 1.5. Since the slag’s nucleation model is homogeneous nucleation at a constant rate, the growth rate of the crystal is mainly determined by the ions’ diffusion control, which can be found in Table 3. When the rotation speed is 200 rpm and 300 rpm, the nucleation method of the crystal is instantaneous nucleation, and the Avrami exponent is close to 1. It indicates that the growth rate of the crystal is still mainly affected by the diffusion rate of the ions in the slag, but the degree of influence gradually decreases with crystallization. The nucleation sites in the remaining molten slag reduce continuously as the crystal progresses [23]. Therefore, *n_n_* decreases continuously between 0 and 1. The interface reaction becomes the main limiting factor of the crystallization of the slag as the rotational speed increases to 400 rpm. It indicates that the effect of agitation on the crystal reduces gradually.

#### 3.5.2. Effect of Shear Stress on the Crystallization Activation Energy of Mold Fluxes

Crystallization activation energy is an energy barrier that must be overcome when the crystals precipitate from mold slags, which is important for reflecting the crystallization performance of mold fluxes. The larger the activation energy of crystallization, the more difficult it is for the crystal to precipitate. The Arrhenius formula [33,34] is used to calculate and analyze the crystallization activation energy of the mold fluxes at different agitation strengths (Equation (6)):

(6) k=Aexp(−Ea/RT)

where *Ea* is the activation energy of crystallization, *A* is the pre-exponential factor, *R* is the gas constant, and *T* is the absolute temperature (notice the unit is K).

Rearranging Equation (6), the value of *Ea* could also be determined by plotting ln*k* versus 1/(*RT*) as in Equation (7):

(7) lnk=lnA−Ea/RT

The crystallization activation energy of molten slags calculated according to Formula (7) at different rotating speeds is shown in Table 5. It can be found that with an increase in the rotating speed, the crystallization activation energy of the crystal gradually decreases, indicating that the energy barrier to overcome for crystallization is getting lower and lower, and the crystal is more easily precipitated. The activation energy decreases significantly from the decrement of the crystallization activation energy, with the rotational speed increasing from 100 rpm to 200 rpm and 200 rpm to 300 rpm, more so than the decrement with the speed increasing from 300 rpm to 400 rpm. It is because the main factor that restricts crystal growth is the diffusion of the ions at the initial stage of crystallization, according to the above studies. The crystallization process is promoted by increasing the diffusion of the ions through the intensification of the rotational speed. Additionally, stirring can produce more nucleation sites and might cause some bubbles to form in the slag, which could form an interface where the crystals are easily precipitated. Meanwhile, the stirring also promotes the heat transfer and increases the supercooling of the slag. All of these factors can promote the crystallization process and reduce the crystallization activation energy. When the rotational speed reaches 400 rpm, the factor that limits the crystallization becomes an interfacial reaction. Therefore, the intensifying effect of agitation on the crystallization is reduced. The growth rate of crystals may even be suppressed by the agitation. The ions participating in crystallization may be detached from the crystal nucleus by the large shear stress.

### 3.6. Effect of Shear Stress on the Lubricating Property of Mold Fluxes

**Figure 9:**
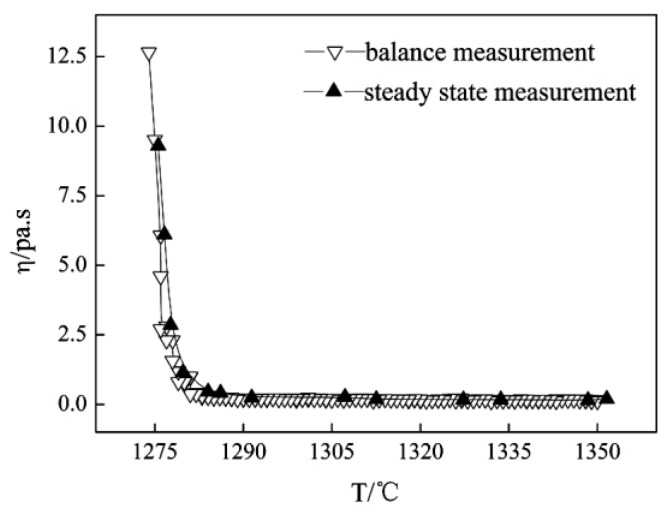
Viscosity–temperature curve of mold flux was obtained by the steady-state and dynamic measurement of the viscosity with no agitation.

**Figure 10:**
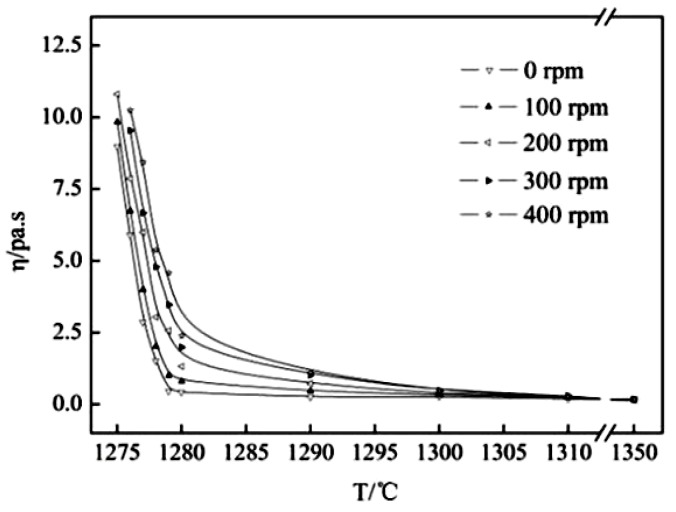
Viscosity–temperature curve of mold flux measured by the equilibrium method with different rotating speeds.

The viscosity–temperature curve of the investigated mold flux measured by the steady method and the balance method with no agitation (0 rpm) is shown in Figure 9. Figure 9 demonstrates that the viscosity–temperature curves obtained by the steady method and the balance method are consistent with each other and the break temperature is similar. Therefore, the balance method can be used to measure the slag viscosity–temperature curve and the break temperature. The measured results under different rotating speeds are found in Figure 10. Figure 10 shows that the viscosity–temperature curve under no-stirring is relatively gentle, and the viscosity increases slightly as the temperature decreases before the break temperature under stirring conditions. However, the viscosity increases sharply as the temperature decreases, with and without stirring, after the break temperature. As the stirring speed increases, the break temperature gradually increases, which is related to the precipitation of crystals in the slag [35]. Table 6 shows the viscous flow activation energy and the break temperature data of slags at different rotational speeds. Table 6 shows that the viscous flow activation energy of the slag increases slightly as the stirring speed increases. The viscous flow activation energy of slags is related to the bond energy of the adjacent ion in molten slag [36,37,38]. The slag structure is rearranged by the crystals, and the crystalline fraction is promoted by the shear stress. Thus, the energy bond increases in the molten slag with agitation. Therefore, the break temperature and viscous flow activation energy are increased by agitation, but the increment of the break temperature and the viscous flow activation energy are small, which is insufficient to deteriorate the lubrication conditions in the casting process. The break temperature will be affected by a sufficient number of crystals precipitated, and the viscosity increase caused by the temperature decrease is the most important factor affecting the break temperature, which is closely related to the solidification temperature of the slags [22,39]. However, the stirring has no effect on the solidification temperature of the slag. Therefore, the continuous casting process can be smooth under different rotating speeds.

## 4. Conclusions

The quantitative effects of different shear stress fields on the crystallization behavior under the isothermal temperature and lubrication properties of the CaO-Al_2_O_3_-SiO_2_-Na_2_O-CaF_2_ slags system were studied. The following conclusions were obtained:

1. The crystallization performance of mold fluxes is promoted by the shear stress, and the quantified crystalline fraction of slag films with different rotating speeds at 1573 K is obtained;
2. The shear stress affects the microscopic morphology of crystals, leading to smaller grain size. However, it has no effect on the crystalline phase of the mold fluxes;
3. The crystallization activation energy reduces under the shear stress through improving the crystallization kinetics of crystals precipitated by the molten slags;
4. The effect of shear stress on the viscosity–temperature curve of the mold fluxes is small. The break temperature and face activation energy slightly increase with the increasing rotational speed, which does not influence the lubrication property in the continuous casting process.

## Figures and Tables

**Table 1:** Chemical compositions of the mold flux (weight percent).

Chemical Compositions	Contents(Weight Percent)
CaO	37.7
SiO_2_	34.3
Al_2_O_3_	5
CaF_2_	15
**Na_2_O**	8

**Table 2:** The crystalline fraction of the slag film under the different stirring intensity for 5 min at 1300 °C (weight percent).

Rotating Speed/rpm	Crystalline Fraction/%	Increment
0	2.7	-
100	10.3	7.6
200	25.1	14.8
300	42.8	17.7
400	47.6	4.8

**Table 3:** Results of the *n* and *k* analysis for isothermal crystallization of mold fluxes under different rotating speeds.

Temperature/K	Rotating Speed/rpm	*n*	ln*k*
1573	100	1.43	−4.33
	200	1.13	−3.16
	300	0.99	−2.25
	400	0.85	−1.56
1568	100	1.45	−4.11
	200	1.22	−3.05
	300	1.01	−2.04
	400	0.89	−1.54

**Table 4:** Value of n for different nucleation and growth mechanisms [31,32].

Crystals Growth Mode	Crystallization Mode	
	Diffusion Controlled	Interface Reaction Controlled
Constant nucleation rate		
Three-Dimensional growth	2.5	4
Two-Dimensional growth	2	3
One-Dimensional growth	1.5	2
Instantaneous nucleation		
Three-Dimensional growth	1.5	3
Two-Dimensional growth	1	2
One-Dimensional growth	0.5	1
Surface nucleation	0.5	1

**Table 5:** Results of crystallization activation energy for the isothermal crystallization of mold fluxes under different rotating speeds.

Rotating Speed/rpm	Crystallization Activation Energy/kJ·mol^−1^	Decrement
100	−13.109	-
200	−72.103	−58.993
300	−137.650	−65.547
400	−154.205	−16.554

**Table 6:** Results of break temperature and viscous flow activation energy of mold fluxes under different rotating speeds.

Rotational Speed/rpm	Break Temperature/°C	Viscous-Flow Activation Energy/kJ·mol^−1^
0	1277	121.602
100	1277	121.797
200	1280	126.978
300	1281	128.771
400	1281	130.449
